# Translation and Validation of the Malay Perceived Stress Scale Modified for COVID-19

**DOI:** 10.21315/mjms2023.30.2.15

**Published:** 2023-04-18

**Authors:** Norhayati Ibrahim, Andrea Wong, Choy Qing Cham, Sin Yee Chu, Clarisse Roswini Kalaman, Ching Sin Siau

**Affiliations:** 1 Centre for Healthy Aging and Wellness (H-Care), Faculty of Health Sciences, Universiti Kebangsaan Malaysia, Kuala Lumpur, Malaysia; 2 Institute of Islam Hadhari, Universiti Kebangsaan Malaysia, Selangor, Malaysia; 3 Centre for Community Health Studies (ReaCH), Faculty of Health Sciences, Universiti Kebangsaan Malaysia, Kuala Lumpur, Malaysia

**Keywords:** validation, perceived stress, COVID-19, Perceived Stress Scale modified for COVID-19, Malaysia

## Abstract

**Background:**

Stress amidst the COVID-19 pandemic is becoming more prevalent. This paper aimed to describe the validation process of the Malay Perceived Stress Scale modified for COVID-19 (PSS-10-C) amongst Malaysian youths.

**Methods:**

The cross-sectional validation study design was employed in this study. In Phase I, the scale was translated into Malay by using the forward-backward method. In Phase 2, principal axis factoring and confirmatory factor analysis were conducted in Study 1 (*n* = 267) and Study 2 (*n* = 324), respectively.

**Results:**

A two-factor solution, comprising ‘distress’ and ‘coping’ domains was derived (cumulative variance = 65.2%) in Phase 2. Concurrent validity evaluated via the Beck Hopelessness Scale revealed a moderate positive correlation (0.528). In Study 2 (*n* = 324), the confirmatory factor analysis showed that the two-factor model achieved acceptable model fit indices, including χ^2^/df ratio = 2.57; root mean square error of approximation (RMSEA) = 0.07; 95% CI = 0.05, 0.09; Tucker-Lewis Index (TLI) = 0.95 and Normed Fit Index (NFI) = 0.94. The Cronbach’s alpha scale score was 0.855 for the study samples.

**Conclusion:**

The Malay PSS-10-C is a valid and reliable scale to be used amongst Malaysian youths.

## Introduction

The coronavirus disease (COVID-19) pandemic (caused by the SARS-CoV-2 virus) is not only a threat to global health, but also has affected the mental well-being of many individuals (1, 2). Due to the exponential spread of the virus to various countries, the World Health Organization (WHO) declared a global health emergency on 12 March 2020 (3). This declaration is a global red flag, indicating that all countries must develop a strategic plan to deal with the new virus spread (4).

In Malaysia, the Prime Minister of Malaysia announced the Movement Control Order (MCO) on 18 March 2020 (5). Due to the consequent social distancing and movement control measures, accompanied by widespread economic and social sequelae as well as fear of the COVID-19 virus, many Malaysians were psychologically affected (6–9). These adverse conditions can increase the stress level of the individuals, especially those who experienced difficulties in coping and adapting (10). Stress due to COVID-19 could possibly be devastating and compounding the psychological pressure already experienced by many individuals (9, 11).

The COVID-19 pandemic has not only psychologically impacted individuals, but also has put a toll on the daily challenges faced by the general population (10). Amongst the student population, first wave of the COVID-19 pandemic in the first 4 to 5 months of 2020 led to major changes in their daily lives (12, 13). Factors such as worrying about their further study plans (6) and the fear of extension of studies (10) may have increased the prevalence of stress symptoms amongst university students, ranging between 22% and 27.6% (14, 15). Past researchers also revealed that working adults experienced stress between 31.6% and 70% during the COVID-19 pandemic (16, 17). This may be due to the vast socioeconomic impact of the pandemic on safety and livelihood of all segments in the society (18).

The Perceived Stress Scale modified for COVID-19, or known as the COVID-19 Pandemic-Related Stress Scale (PSS-10-C) was adapted from the Perceived Stress Scale-10 (PSS-10). Cohen et al. (19) created the Perceived Stress Scale (PSS), which evaluates the global view of stress by measuring emotions and feelings in the past month. The wide usage of this scale can be attributed to its simplicity and free availability for research or academic purposes. The PSS-10-C was later adapted by Pedrozo-Pupo et al. (20) amid the COVID-19 pandemic. The PSS-10-C presented a two-dimensional structure and acceptable internal consistency reliability (21). Past studies stated that the PSS-10-C has an added advantage as the scale demonstrated good reliability when it was conducted amongst university students (20–23).

To date, a validated COVID-19-related stress scale in the Malay language is unavailable for stress level assessment in the Malaysian setting during and post-COVID-19 pandemic. A widely used scale in Malaysia, the Fear of COVID-19 Scale that was developed by Ahorsu et al. (24) and validated in the Malay language by Pang et al. (25), was aimed to assess the general population’s fear of COVID-19. However, this scale did not assess or evaluate individuals’ stress level due to the COVID-19 pandemic. Therefore, this study is aimed to validate the Malay version of PSS-10-C and evaluate its psychometric properties for the perceived stress assessment due to COVID-19 amongst Malaysians.

## Methods

### Study Design

**Figure 1:**
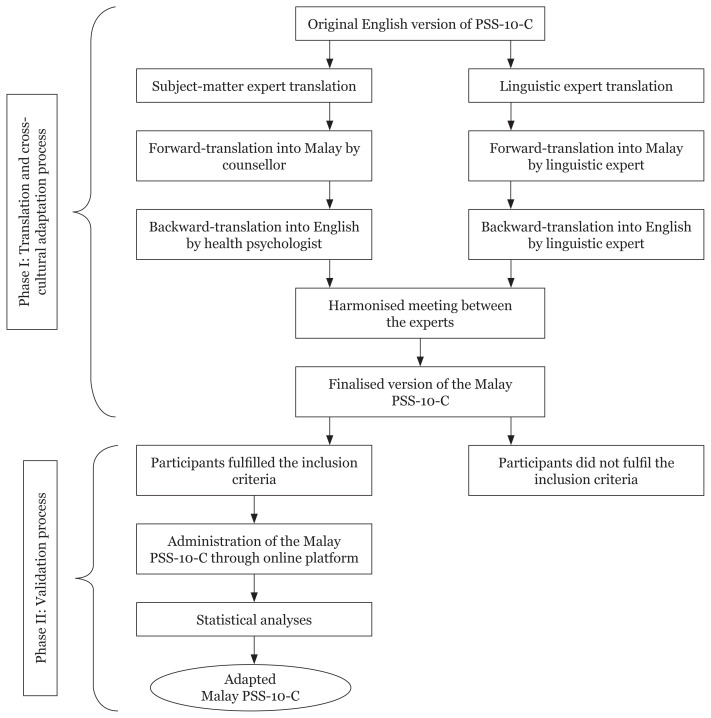
Flowchart for the translation and validation process of the English PSS-10-C into Malay PSS-10-C

This cross-sectional study conducted in Malaysia was to establish the validity and reliability of the Malay version of PSS-10-C within the Malaysian context. he flowchart for the translation and validation process of the English PSS-10-C into Malay PSS-10-C is shown in Figure 1.

#### Phase I: Translation and Adaptation Process

The PSS-10-C was modified by Pedrozo-Pupo et al. (20) from the PSS-10 (26), to measure the perception of stress during the COVID-19 pandemic amongst Colombian citizens. The PSS-10-C consisted of 10 items and participants responded on a 5-point Likert scale (0 = Never, 4 = Always). A bifactor model was indicated which represented perceived distress and perceived coping. Therefore, a total score would be calculated, in which higher scores represent higher levels of perceived stress. The scale demonstrated good internal consistency reliability amongst participants with a Cronbach’s alpha coefficient of 0.86 (21).

To translate the English PSS-10-C into Malay language, the forward-backward translation method was conducted by linguistic and subject matter experts. The subject-matter experts consisted of a counsellor who did the forward-translation into Malay language and a health psychologist who did the backward-translation into the English language. To ensure the face validity of this scale, a harmonisation meeting was held to combine the two versions of translated questionnaire and finalise the questionnaire. During the adaptation process to finalise the Malay version of scale, researchers considered the contextualised meaning attached to a construct, barriers in linguistics comprehension, and possible interpretations of the translated scale (27).

#### Phase II: Validation Process

## Participants

This study involved Malaysian youths aged from 18 years old to 40 years old. For Study 1, participants were young adults who have recently graduated, were currently working or were looking for jobs. For Study 2, Malaysian students from local and private universities were recruited. Individuals were eligible for inclusion if they were Malaysians, aged between 18 years old and above, sufficiently literate in Bahasa Malaysia and able to provide an informed consent for the study. An additional inclusion criterion of being a university student enrolled in one of the tertiary education institutions in Malaysia was added for the participant recruitment in Study 2. Those unable or unwilling to provide an informed consent were excluded.

For validation, the questionnaire was adhered to the rule of thumb in recruiting 2–20 participants per item (28–29) with minimum of 250 participants (31). Therefore, the current study targeted to recruit a minimum of 250 participants per study, which was equivalent to 25 participants per item.

### Measures

Apart from the PSS-10-C, the Beck Hopelessness Scale (BHS) was used in Phase 2 of the study. The BHS was constructed by Beck et al. (31) as a self-reported questionnaire to measure the negative attitudes regarding one’s future. The BHS consisted of 20 true-false items categorised into three factors: i) feelings about the future, ii) loss of motivation, and iii) future expectations. The total score for BHS ranged from 0 to 20; higher scores denoted higher levels of hopelessness. The BHS had good internal consistency reliability estimates with Cronbach’s alpha ranging from 0.82 to 0.93 (32). The BHS-Malay was validated amongst the Malaysian population and reported good psychometric properties with internal consistency reliability estimates that ranged from 0.71 to 0.91 (33). Convergent validity, construct reliability and Cronbach’s alpha for the BHS-Malay was found to be 0.60, 0.75 and 0.74, respectively, when tested on 500 Malaysian undergraduates (34).

## Procedures

Convenience sampling was used to collect data and the online survey link was distributed through a variety of social media platforms and e-mail lists. Study 1 was carried out amongst non-student youths with the objective of validating the construct and concurrent validity of the scale. Study 2 was carried out amongst university students to further test the model fit of the factor structure obtained in Study 1. Participation in the study was voluntary and strictly confidential without any identifier being used in the questionnaire. All pertinent information concerning the study was written on the questionnaire, enabling participants get a comprehensive understanding concerning the study before voluntarily participated in the study. Since the questionnaire was distributed online, participants were afforded sufficient time to consider their participation and fill the questionnaire unrushed. Then, an informed consent was obtained from the participants before proceeding to the next part of the questionnaire. Participants were required to fill a set of questions which consisted of three main sections: i) demographic details including age, gender, race, monthly household income and educational level, ii) Malay version of PSS-10-C and iii) Malay version of BHS. The questionnaire took approximately 20 min and participants were free to withdraw from the study if any discomfort arose during the study.

## Statistical analysis

Statistical analyses were conducted by using the IBM SPSS Statistics for Windows, version 24.0 (35) and the IBM SPSS Amos, version 20.0 (36). In Study 1, exploratory factor analysis, involving principal axis factoring (PAF) by using varimax rotation was employed to determine the latent constructs that influenced how the participants answered the questions (37). Pearson correlation coefficient was employed to assess the concurrent validity of the Malay PSS-10-C with BHS. Items with < 0.40 factor loading and < 0.20 communality would be excluded from further analysis.

In Study 2, model fit indices were evaluated via a confirmatory factor analysis, which included the chi-square-value/degree of freedom (χ^2^/df < 3.0) (38), Normed Fit Index (NFI ≥ 0.95), Tucker-Lewis index (TLI ≥ 0.95) (39), parsimonious normed fit index (PNFI ≥ 0.50) (40) and root mean square error of approximation (RMSEA ≥ 0.08) [41–42].

To examine internal consistency reliability of the scale, Cronbach’s alpha coefficient was employed. Items that contributed to Cronbach’s alpha of more than 0.70 were retained, whereas items that contributed to a low coefficient would be excluded from further analysis (43, 44). The examination of internal consistency reliability estimates was conducted on the total pool of Study 1 and Study 2 participants.

## Results

### Participant Characteristics

A total of 591 participants responded to the questionnaire. Participants from Study 1 comprised non-student youths (*n* = 267 [45.2%]) and participants from Study 2 were university students (*n* = 324 [54.8%]). The demographic characteristics of the participants from Study 1 and Study 2 are reflected in Table 1.

### Study 1: Validity Analysis

Exploratory factor analysis by using principal axis factoring (PAF) was conducted. The minimum amount of data required for factor analysis was met, which was 26 cases per questionnaire item. Factorisation by using varimax rotation, revealed an acceptable Kaiser-Meyer-Olkin measure of sampling adequacy (KMO = 0.87), which was above the recommended value of 0.60. Bartlett’s test of sphericity was significant, χ^2^(45) = 1302.82, *P* < 0.001. The diagonals of the anti-image correlation matrix of all items were above 0.50 (range of 0.79–0.91) and thus, supported the inclusion of each item in the factor analysis. All items had communalities ≥ 0.20 (range of 0.35–0.74) (Table 2), suggesting reasonable factorability. Given these overall indicators, factor analysis was performed on all 10 items of the Malay PSS-10-C.

PAF examined the solutions for one and two factors. The two-factor solution was preferred, as it explained a higher percentage of cumulative variance (65.2%) as compared to the one-factor solution (44.5%), which was more than the recommended cumulative variance of 60%. Factor 1 (consisted of items 1, 2, 3, 6, 9 and 10) accounted for 44.5% of the variance and Factor 2 (consisted of items 4, 5, 7 and 8) accounted for another 20.7% of the variance. All items had primary loadings of above 0.40 (range of 0.59–0.84) (Table 2). The correlation between Factor 1 and Factor 2 was *r* (267) = 0.289, *P* < 0.001.

#### Concurrent Validity

Concurrent validity was analysed by correlating PSS-10-C with the BHS. Results showed that the scales correlated significantly, *r* (267) = 0.528, *P* < 0.001.

### Study 2: Confirmatory Factor Analysis

Based on the one-factor and two-factor solutions obtained through the PAF of Study 1, confirmatory factor analyses to test the model fit for both solutions were performed. Results showed that the two-factor solution demonstrated a better fit value of χ^2^/df ratio = 2.57, which was within the < 3.00 cut off suggested by Carmines and McIver (38) and was far superior than the one-factor solution value of 9.28. The two-factor model also demonstrated a goodness-of-fit according to the RMSEA (0.07), which showed a reasonable error of approximation (40) and PNFI of 0.70, which was above the recommended value of ≥ 0.50. The TLI index met the required cut-off (0.95) while the NFI index (0.94) was close to the recommended cut-off of ≥ 0.95 (Table 3).

### Reliability Analysis

For Study 1, internal consistency reliability estimates for the scale score was α = 0.855 and α coefficient for Factor 1 and Factor 2 were 0.898 and 0.796, respectively. For Study 2, internal consistency reliability estimates for the scale score was α = 0.855, and α coefficients for Factor 1 and Factor 2 were 0.875 and 0.765, respectively. The total internal consistency reliability estimates for the scale based on the combined data of Study 1 and Study 2 was *α* = 0.859 and the α coefficients for Factor 1 and Factor 2 were 0.891 and 0.785, respectively. Furthermore, the Cronbach’s alpha coefficients were 0.855 and 0.850 amongst non-student youths and student samples, respectively. Descriptive analysis of the questionnaire items and factors are shown in Table 4. The least endorsed item was Item 8 “I have felt that I have everything under control in relation to the epidemic,” at 22.2%, while the most endorsed item was Item 1 “I have felt affected as if something serious will happen unexpectedly with the epidemic” at 44.5%. All items had more than 20% of the respondents who chose the middle option ‘Occasionally’.

## Discussion

This study aimed to examine the validity and reliability of the Malay PSS-10-C. The major findings were that the Malay PSS-10-C consisted of two domains, and the internal consistency reliability estimates were *α* = 0.855, 0.875 and 0.765 for the overall scale score, Factor 1 and Factor 2, respectively. Confirmatory factor analysis showed that the two-factor solution had better model fit as compared to a one-factor solution.

In terms of the scale validity, factor loadings and communalities of the Malay PSS-10-C were higher than the cut-off of 0.40 and 0.20, respectively (45, 46). Nine out of 10 items in the Malay PSS-10-C loaded into the same domain as the original PSS-10-C validated by Campo-Arias et al. (2). However, one item (Item 6: “I have felt unable to cope with the things I have to do to control the possible infection”) which was in the ‘coping’ domain in the original Colombian questionnaire had loaded in the domain of ‘distress’ in the Malay PSS-10-C. This showed that the constructs of ‘distress’ and ‘coping’ in the PSS-10-C were similar between Colombian and Malaysian participants. Being ‘unable to cope’; however, signified a more distressed state for Malaysian study participants. As compared to the original PSS-10-C, the Malay PSS-10-C demonstrated a higher percentage of total cumulative variance explained (65.2%), which was 8.6% higher than the original Colombian PSS-10-C. It was interesting to note that the factor loadings and the two-factor solution of the Malay PSS-10-C was comparable with the Malay PSS-10 scale (47), which also reported two factors with items 1, 2, 3, 6, 9 and 10 loading into Factor 1 and items 4, 5, 7 and 8 loading into Factor 2. The low correlation between the two factors (0.289) in the Malay PSS-10-C further indicated suitability of the two-factor solution.

Regarding the concurrent validity of the Malay PSS-10-C, the scale was moderately and positively correlated with the Beck Hopelessness Scale, indicating that the PSS-10-C could be used to relate to hopelessness during the COVID-19 pandemic. A positive relation between hopelessness and stress was also demonstrated in another study (48), and may be explained by the fact that greater perceived stress may generate pessimism about the future.

The model fit indices of the two-factor solution for the PSS-10-C was within acceptable ranges for both the absolute (RMSEA) and relative (TLI and PNFI) fit indices. The χ^2^/df was below 3, indicating an acceptable fit between the hypothesised model and the sample data (38). In addition, the high PNFI value suggested that the two-factor solution was a parsimonious model. Overall, the model fit of the Malay PSS-10-C performed better than the original Colombian scale, which recorded fit indices of χ^2^/df=8.7, RMSEA = 0.08, CFI = 0.93 and TLI = 0.91 (21). Perhaps there were cultural differences regarding perceived stress of the COVID-19 pandemic between the two countries, which were as yet to be determined.

Finally, this study showed that the Malay PSS-10-C demonstrated acceptable internal consistency reliability of Cronbach’s alpha ≥ 0.70 for the total scale as well as its domains. Apart from that, the scale was also reliable when tested amongst the non-student young adult and student samples of Study 1 and Study 2, demonstrating its reliability across the two groups. The results indicated that across the student and non-student youth samples, these items measured the same construct or content and was a reliable scale for use in the two groups. Therefore, the scale was a reliable instrument to be used in the Malaysian youth sample.

### Strengths and Limitations

This is the first study to adapt and validate a PSS-10-C amongst the Malaysian population. The scale was applicable to be used amongst students and non-student youths. However, the study results may not be representative of Malaysians as almost all respondents were from the Malay ethnic group. Besides that, the female-to-male ratio of respondents was not representative of the Malaysian population. The heterogeneity of respondents in this study was partially achieved, as respondents involved were youths from different occupational backgrounds. However, participants older than 40 years old were not sampled. Nevertheless, due to the brevity, reliability and validity of the Malay PSS-10-C, the scale would still be deemed useful in assessing individuals’ perceived stress related to COVID-19 pandemic in Malaysia. To make comprehensive comparisons and increase generalisability of the Malay PSS-10-C, studies in the future will need to employ different types of population and setting to further explore and improve the psychometric rigor of the scale.

## Conclusion

The Malay PSS-10-C scale demonstrated acceptable validity and reliability across student and non-student youth samples. The two-factor model achieved a cumulative explained variance of 65.2%. The two-factor solution of ‘distress’ and ‘coping’ domains demonstrated better model fit as compared to a one-factor solution, and was consistent with the original PSS-10-C scale and the Malay PSS-10 scale. Model fit indices indicated good model fit for both absolute and relative fit indices. Internal consistency reliability was acceptable for the summed scale score and across its two domains. Therefore, the Malay PSS-10-C is a valid and reliable instrument to be used amongst Malaysians to measure perceived stress as a result of the COVID-19 pandemic.

## Figures and Tables

**Table 1:** Demographic characteristics of study participants (*N* = 591)

Variable	Study 1 (*n* = 267)	Study 2 (*n* = 324)	Total (*n* = 591)

	*n* (%)	*n* (%)	*n* (%)
Age (years old)			
18–25	88 (33.0)	302 (93.2)	390 (66.0)
26–30	105 (39.3)	18 (5.6)	123 (20.8)
31–35	48 (18.0)	2 (0.6)	50 (8.5)
36–40	26 (9.7)	2 (0.6)	28 (4.7)
Gender			
Male	30 (11.2)	49 (15.1)	79 (13.4)
Female	237 (88.8)	275 (84.9)	512 (86.6)
Ethnicity			
Malay	190 (71.2)	191 (59)	381 (64.5)
Bumiputera Sabah	36 (13.5)	21 (6.5)	57 (9.6)
Bumiputera Sarawak	9 (3.4)	5 (1.5)	14 (2.4)
Chinese	22 (8.2)	83 (25.6)	105 (17.8)
Indian	7 (2.6)	24 (7.4)	31 (5.2)
Others	3 (1.1)	0 (0)	3 (0.5)

**Table 2:** Explained variance, factor loadings, and communalities based on a principal components analysis with varimax rotation for 10 items of the Malay PSS-COVID-10 questionnaire

Item No.	Item	Explained variance (%)	Factor loading	Communality
	Total	65.2		
	**Factor 1: Distress**	44.5		
2	I have felt that I am unable to control the important things in my life due to the epidemic. *Saya rasa tidak mampu mengawal perkaraperkara penting…*		0.842	0.74
3	I have been nervous or stressed by the epidemic. *Saya rasa gementar atau tertekan …*		0.793	0.66
10	I have felt that the difficulties accumulate in these days of the epidemic and I feel unable to overcome them. *Saya rasa kesulitan semakin menimbun semasa keadaan wabak ini ...*.		0.776	0.64
1	I have felt affected as if something serious will happen unexpectedly with the epidemic. *Saya rasa terkesan seolah-olah sesuatu yang serius akan berlaku* ...		0.754	0.58
9	I have been upset that things related to the epidemic are out of my control. *Saya rasa kecewa kerana segala perkara yang berkaitan dengan wabak …*.		0.711	0.51
6	I have felt unable to cope with the things I have to do to control the possible infection. *Saya rasa tidak mampu menangani pelbagai perkara yang harus saya lakukan …*.		0.691	0.48
	**Factor 2: Coping**	20.7		
7	I have felt that I can control the difficulties that could appear in my life due to the infection. *Saya rasa bahawa saya mampu mengawal kesulitan yang mungkin muncul ...*.		0.799	0.67
5	I have felt that things are going well (optimistic) with the epidemic. *Saya rasa bahawa segala perkara berjalan dengan baik (optimistik) ..*.		0.698	0.53
8	I have felt that I have everything under control in relation to the epidemic. *Saya rasa bahawa saya mampu mengawal segala perkara ..*.		0.693	0.50
4	I have been confident about my ability to handle my personal epidemic related problems. *Saya rasa yakin dengan kemampuan saya untuk menangani masalah peribadi* …		0.589	0.35

**Table 3:** Goodness-of-fit indicators for the 1- and 2-factor solutions for the 10-item Malay PSS-10-C questionnaire (*N* = 324)

Model	χ^2^ (df)	χ^2^/df^a^	NFI^b^	TLI^c^	PNFI^d^	RMSEA^e^ (95% CI)^f^
1-factor	324.85 (35)	9.28	0.76	0.72	0.59	0.160 (0.144, 0.176)
2-factor	87.47 (34)	2.57	0.94	0.95	0.71	0.070 (0.052, 0.088)

Notes:

a degree of freedom;

b Normed Fit Index;

c Tucker-Lewis Index;

d Parsimonious Normed Fit Index;

e root mean square error of approximation;

F Confidence Intervals

**Table 4:** Descriptive statistics for the Malay PSS-10-C questionnaire

Domain	Mean (SD)	Min (Max)	Range	α	Item no.	Never/ Hardly ever *n* (%)	Almost always/ Always *n* (%)	Occasionally *n* (%)
1. Distress	12.77 (6.13)	0 (24)	0–24	0.891	1	152 (25.5)	265 (44.5)	178 (29.9)
					2	20.5 (34.5)	220 (37.0)	170 (28.6)
					3	176 (29.6)	242 (40.7)	177 (29.7)
					6	197 (33.1)	204 (34.3)	194 (32.6)
					9	175 (29.4)	244 (41.0)	176 (29.6)
					10	199 (33.4)	209 (35.1)	187 (31.4)
2. Coping	8.35 (3.41)	0 (16)	0–16	0.785	4	209 (35.1)	168 (28.2)	218 (36.6)
					5	156 (26.2)	226 (38.0)	213 (35.8)
					7	215 (36.1)	148 (24.9)	232 (39.0)
					8	132 (22.2)	255 (42.9)	208 (35.0)
